# Role of Talc Modulation on Cytokine Activation in Cancer Patients Undergoing Pleurodesis

**DOI:** 10.1155/2012/806183

**Published:** 2012-03-07

**Authors:** Yehuda Schwarz, Alex Star

**Affiliations:** Department of Pulmonary Medicine, Tel-Aviv Sourasky Medical Center, Sackler Faculty of Medicine, Tel Aviv University, Tel-Aviv 64239, Israel

## Abstract

We investigate the mechanism of talc pleurodesis (TP) in 20 patients with recurrent malignant pleural effusion and 10 patients with nonmalignant pleural effusions. We measured IL-8 levels before and 6 h after TP and find a significant threefold increase (2.26 ng/mL ± 0.7 to 6.5 ng/mL 0.1), which explains the recruitment of inflammatory cells in these patients. We hypothesize that TP is enable by stimulating the mesothelial cells (MS) to secrete FGF. A significant tenfold increase in FGF-b (0.05 ng/mL ± 0.02 to 0.44 ng/mL 0.6) was seen 24 h after talc instillation (*P* < 0.04). In order to examine whether FGF-b is secreted by MS cells, MS recovered from CHF patients with recurrent pleural effusions were cultured for 48 h in the presence or absence of increasing concentrations of talc (from 100 ng/mL to 1 mg/mL). They produced significant levels of FGF-b in a dose dependent manner (*P* < 0.005). We hypothesized that a successful pleurodesis involves an early enhanced recruitment of inflammatory cells through a rise of IL-8 followed by enrollment of fibroblasts from the submesothelial space through increased mesothelial FGF-b production.

## 1. Introduction

Recurrent pleural effusion in cancer patients is a common problem that significantly affects their quality of lives. Several palliative treatment options are available for malignant recurrent pleural effusion in cancer patients who are not responding to chemotherapy, repeated thoracocentesis, or chemical pleurodesis. The most widely used pleurodesis technique is based on the instillation of sclerosing agents into the pleural space. The therapeutic goal in the management of symptomatic patients with malignant pleural effusions is to achieve effective pleural sclerosis. The aim of the sclerosant agent is to irritate both pleurae and to induce mesothelial cell sloughing and subsequent formation of adhesions between the parietal and visceral surfaces. Although the agent must have irritant attributes, it needs to limit and even obviate adverse effects to the patient. Several sclerosing agents are used in clinical practice, among them doxycycline, minocycline, tetracycline, bleomycin, cisplatin, etoposide, fluorouracil, interferon-*β*, and corynebacterium parvum. Talc has minimal long-term adverse side effects and it was shown to be the most effective agent for preventing recurrences [1]. The mechanisms of pleurodesis have not yet been entirely understood. Diffuse pleural inflammation and fibrin deposition have been considered influential in the success of pleural symphysis [2]. In animal studies, histologic analysis of talc-treated pleurae [2] showed neutrophilic predominance at 24 h, followed by mononuclear infiltration into the subpleural connective tissue matrix and peripheral airspace of the lung. At day 7, the mononuclear infiltrate was accompanied by fibroblasts and collagen. Rodriguez-Panadero et al. [3] showed a significant increase of neutrophil count in the pleural fluid from patients whose pleurodesis treatment was successful. Is not clear how the presence of increased neutrophils in the pleural space contributes to the success of pleural symphysis, but it does indicate that the first step of neutrophilic recruitment is critical. The arrival of inflammatory phagocytic cells is mediated via the release of chemotactic cytokines by activated mesothelial cells [4]. Indeed, the ability of mesothelial cells to release chemokines following stimulation by LPS, IL-1, or TNF-*α* has been reported [4]. Pleural effusions secondary to various diseases are associated with the presence of different inflammatory cells, but the formation of pleural adhesion is slow compared to adhesion following the instillation of talc and other sclerosing agents. Tetracycline, which was widely used in the past as a sclerosing agent, increases IL-8 and neutrophil predominance and release of growth-factor-like activity for fibroblasts [5, 6]. The deposition of efficient fibrin within the pleural space in malignant effusions to induce pleurodesis is scanty. In contrast, effusions induced by injury and inflammation the deposition of fibrin has been often demonstrated [7–10]. It was postulated by Kuwahara et al. that the pathogenesis for pleural fibrosis after pleural injury is through fibroblast recruitment to the pleural damage sites by mesothelial cells [11]. Those researchers used fibronectin as the chemotaxin and they showed a progressive time-dependent increase in fibroblast chemoattractant activity by pleural mesothelial cell throughout 96 h cultures. This secretion of a mesothelial cell-derived fibroblast chemoattractant may play a role in the response of the pleurae to injury and in the pathogenesis of pleural fibrosis [11]. Fibroblast fibrinopeptides during fibrin formation stimulate local mesothelial regeneration and proliferation as shown by Griffith et al. [12]. Fibrin and collagen deposition in the pleural connective tissue and in intrapleural adhesions was reported by Hurewitz et al. after instillation of tetracycline and doxycycline for pleurodesis [13]. They showed also that fibroblasts were the predominant inflammatory cells at autopsies after 2 weeks of successful pleurodesis in rabbits. Enhanced proliferation of fibroblasts following asbestos exposure [14] and tetracycline [15] have also been reported.

The mechanisms of talc pleurodesis have yet to be delineated, raising a number of unanswered questions. Is the role of talc in the pleurodesis process limited to an irritant (foreign body) effect that solely induces inflammation or does it have a direct effect on mesothelial and fibroblasts cells? Is the activation of mesothelial cells by the inflammatory process critical to the recruitment and proliferation of fibroblasts, and are the mesothelial cell responsible for fibroblast proliferation? Do mesothelial cells from cancer patients react to talc the same degree as mesothelial cells from normal subjects?

### 1.1. Clinical Relevance

During the evolution of the disease, cancer patients suffer from recurrent pleural effusion that endangers their lives and significantly reduces their quality of life. They are in need of medical support and frequently require immediate treatment. Although talcage pleurodesis is effective, it causes side effects and morbidity. By understanding the fibrotic process produced by talc, we will be able to prevent most of those side effects and lower the risk of injury to the already compromised health of these patients. 

We hypothesized that successful induction of pleurodesis involves a cascade mechanism in which there is early enhanced recruitment of inflammatory cells to the pleural space through secretion of chemokines (mainly IL-8). This inflammatory “soup” allows enrollment and proliferation of fibroblasts from the submesothelial space to the plural space by secretion of fibroblast growth factor production by the mesothelial cells. These steps are induced directly and primary by talc.

## 2. Methods

### 2.1. Clinical Protocol

#### 2.1.1. Study Population

20 patients with recurrent malignant pleural effusions and 10 patients with nonmalignant pleural effusions. Patients are followed during their hospitalization for the talcage procedure and at the outpatient pulmonary and oncology clinics after discharge. Chest X-rays are performed before and shortly after talcage, and before discharge. Pulmonary function tests are also performed before and after talcage. A clinical chart is used to track any failure of pleurodesis induction.

#### 2.1.2. Thoracocentesis

 Diagnostic thoracocentesis is performed under local anesthesia, using the Bard*I-Cath intravenous placement unit (Bard Ltd., UK) No. 16 connected through a three-way stopcock to a syringe.

#### 2.1.3. Pleural Fluid Examination

 Fluid is examined routinely for cytology, levels of glucose, LDH, protein, cholesterol, and cultures. Pleural fluids from the diagnostic thoracocentesis are collected immediately before talcage, and at 6 h and 24 h after Talcage and frozen at −70°C until cytokine measurement.

#### 2.1.4. Talcage Slurry Procedure

 Instillation of talc (slurry procedure) via tube thoracostomy (Sherwood Medical, Ireland) is performed after full expansion of the lung. Two 2 g of certified USP asbestos-free talc (Biolabs, Israel) sterilized at 150°C is mixed in 100 mL of normal saline solution under sterile conditions, and instillation is performed through the tube thoracostomy at bedside. The tube is clamped for 2 h and the patient undergoes rotational maneuvers. Drainage monitoring and volumes are recorded until drainage falls below 150 mL/24 h at which point the thoracostomy tube can be extracted [16]. 

### 2.2. *In Vitro* Protocol

#### 2.2.1. IL-1 Mediation

The following experiment is designed to clarify whether IL-8 production is mediated by primary IL-1 activation. Cells recovered from pleural thoracocentesis are cultured for 24 h in 1640 RPMI 10% FBS in the presence or absence of IL-1 receptor antagonist (1 *μ*g/mL/10^6^ cells) and talc (1 *μ*g/mL/10^6^ cells). Supernatants are collected for IL-8 measurements.

#### 2.2.2. Talc Studies

Normal mesothelial cells recovered by thoracocentesis from patients with intractable congestive heart failure are cultured at equal concentrations in 1640 RPMI 20% FBS until confluence. Cell viability is tested by trypan blue dye exclusion. Cytospin preparations of pleural fluid cells are performed by counting the cells before culture by cytospins and reconstituting them at a concentration of 7.5 × 10^−5^ mix-cell/mL in RPMI-20% FCS. They are exposed to graded concentrations of talc in serum-free medium at increasing concentrations from 100 ng/mL to 1 mg/mL for 24 h in order to establish a dose-response curve. Supernatants for measuring cytokines and growth factors are collected after centrifugation to remove excess talc and cells.

#### 2.2.3. Cytokine Measurement

IL-8 and human fibroblast growth factor levels are quantified by sandwich-type enzyme-linked immunoassays ELISA (R & D systems, USA).

For the IL-8 and hFGF ELISAs, flat-bottomed 96-well microtiter plates are coated with an excess murine monoclonal antibody to either IL-8 or hFGF. The pleural fluids from the diagnostic thoracocentesis and supernatants from the mesothelial cell culture medium stimulated by serial dilutions of talc are then added. After incubating, any unbound protein is removed by washing with phosphate-buffered saline (PBS) and an enzyme-linked polyclonal antibody specific to either IL-8 or hFGF is added. The antibody binds the antigen that had been immobilized during the first incubation period. Substrate solution is added to the wells after another washing with PBS,. The presence of IL-8 or hFGF is quantified by comparing the optical density (OD) of the samples with the standard curve.

### 2.3. Statistical Analysis

Data were analyzed with the SigmaStat statistical software package (Apple Computer, Cupertino, CA, USA) and expressed as mean ± SD. The differences between the group means were analyzed by analysis of variance (ANOVA), with use of the Student-Newman-Keuls test. Data were considered statistically significant at *P* < 0.05.

Isolated pleural fluid cells are characterized as pleural mesothelial cells (PMCs). In general, approximately 500 mL of centrifuged transudative pleural fluid yields 12 confluent 3.5 cm diameter Petri dishes of mesothelial cells within 5 to 12 days when cultured as described. The mesothelial cells were positively stained for vimentin, cytokeratin, and hyaluronic acid mucin [17]. Cell morphology was defined by phase-contrast microscopy as having a cobblestone pattern, and numerous microvilli were noted on transmission electron microscopy. All cells were utilized at the second passage in 3.5 cm diameter Petri dishes.

## 3. Results

### 3.1. IL-8 Production in Malignant Pleural Effusion

**Figure 1:**
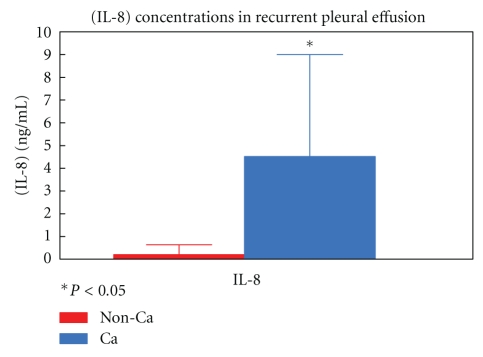
IL-8 concentrations from noncancer noninfectious recurrent pleural effusion compared to malignant pleural effusions.

We found that pleural fluid obtained from patients suffering from recurrent malignant pleural effusion had elevated IL-8 levels (4.52 ng/mL ± 4.4) compared to the pleural fluid obtained from noncancer patients suffering from nonmalignant, noninfectious pleural effusion (0.2 ng/mL ± 0.4) (*P* < 0.05) (Figure 1).

### 3.2. Talc Stimulates IL-8 Secretion at Early Steps of the Pleurodesis Process

**Figure 2:**
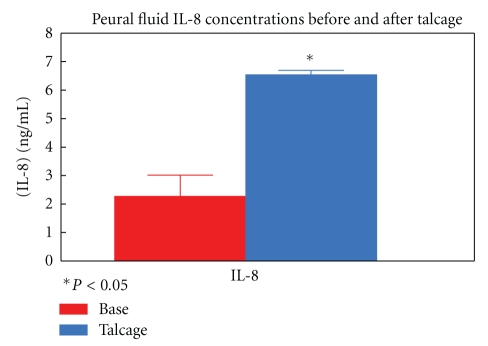
Pleural IL-8 concentrations before and after 6 h from talcage.

The IL-8 levels in malignant pleural effusion showed an early (6 h) significant threefold increase due to talc instillation (2.26 ng/mL ± 0.7 to 6.5 ng/mL 0.1). (Figure 2).

### 3.3. Talc Stimulates Confluent Mesothelial Cells to Release C-X-C Chemokine-IL-8

PMC cultures were stimulated with varying doses of talc (2 to 64 mg/cm^2^) for 24 h in tissue culture plates. Cell viability was documented by trypan blue dye exclusion and vi-intensity character-visual inspection with phase-contrast microscopy. Viable cells were expressed as the percent viable cells of all cells. The PMC viability decreased with increasing talc concentration. PMC viability at a talc concentration of 64 mg/cm^2^ was about 75%. Cultured mesothelial cells released small amounts.

**Figure 3:**
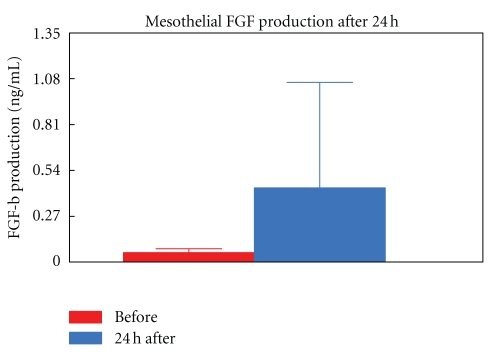
FGF-b production levels baseline and after talc instillation.

There was a significant (*P* < 0.04) tenfold increase in FGF-b levels (0.05 ng/mL ± 0.02 to 0.44 ng/mL 0.6) 24 h after talc instillation (Figure 3).

**Figure 4:**
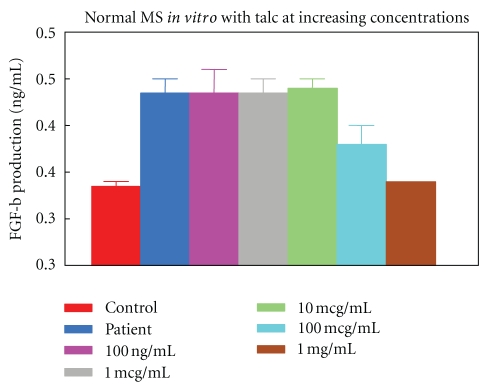
Measurements of FGF-b production levels by Normal mesothelial (MS) cells cultured *in vitro* in the presence of talc at increasing concentrations.

Moreover, normal mesothelial cells were cultured *in vitro* with talc in order to determine whether the mesothelial cells are the ones responsible for the rise in FGF-b levels after talcage: they produced significant levels of FGF-b in a dose-dependent manner (*P* < 0.005) (Figure 4).

## 4. Discussion

In this work, we demonstrated cytokine IL-8 elevation in early stages of talc pleurodesis compared to basic fibroblast growth factor (bFGF) increases in later phases. We also showed that pleural mesothelial cells were either partially or completely responsible for both IL-8 and bFGF elevation.

Current thinking of chemical pleurodesis [18–20] suggests that an acute inflammatory reaction is initiated in the pleural space following the administration of talc, with an influx of neutrophils and mononuclear cells associated with an increase in interleukin-8 and other cytokines in pleural fluids. During the next step, pleural mesothelial cells secrete significant amounts of bFGF that stimulate fibroblast proliferation. The fibroblasts synthesize collagen, resulting in pleural adhesions between visceral and parietal pleurae, and bFGF simultaneously promotes secretion of TGF-*β* and VEGF that accentuates fibrosis.

Our results demonstrate that pleural IL-8 levels from patients with malignant pleural effusion were higher compared to IL-8 levels in patients with nonmalignant, noninfectious pleural effusion (*P* < 0.05). We also reported a three-fold increase of IL-8 at 6 hours after talcage (*P* < 0.005). A number of investigations have shown that mesothelial cells actively contribute to the acute inflammatory process in talc pleurodesis. Genofre et al. [21] evaluated submicroscopic features of active pleural remodeling associated with talc pleurodesis and stated that talc acutely induces a prominent injury to the mesothelial cells.

The mesothelial cells exposed to talc can actively produce proinflammatory cytokines, such as IL-8 [18, 22], VEGF [18], and bFGF [20]. Marchi et al. [19] demonstrated that WBC, neutrophil percentage, and IL-8 levels were increased in the first 24 h after talc exposure. Acencio et al. [23] investigated the acute response of rabbit pleural mesothelial cells challenged with talc. Cultured rabbit pleural mesothelial cells were exposed to and assessed for the production of IL-8, VEGF, and TGF-*β*. At 6 h, the IL-8, VEGF, and TGF-*β* levels produced by talc-exposed mesothelial cells increased significantly and remained elevated for up to 48 h. The investigators concluded that pleural mesothelial cells may actively mediate the primary inflammatory pleural response in talc-induced pleurodesis injection, whereas VEGF and TGF-*β*1 levels were initially lower and increased with time. Therefore, our results confirm the data of other investigators on early involvement of cytokine IL-8 in the talcage procedure that induces recruitment of inflammatory cells in this process.

We hypothesized that a successful pleurodesis induction involves an early enhanced recruitment of inflammatory cells through a rise of pleural IL-8 concentrations followed by enrollment of fibroblasts from the submesothelial space through increased mesothelial bFGF production. We found a ten-fold increase of the bFGF level in pleural fluid 24 hours after talcage that confirms the above hypothesis.

bFGF has been characterized as a key factor in successful pleurodesis as well as in the formation of pleural effusions. It is a member of the FGF family. This group of molecules, similarly to the TGF*β* superfamily, are pleiotropic regulators of cell responses that are involved in a diverse range of biological functions, including proliferation, differentiation and wound healing. Like TGF*β*, bFGF has also been implicated in pleural fibrosis and can stimulate mesothelial cell proliferation *in vitro* and *in vivo*.

We speculate that talc induces pleurodesis-fibrosis by direct stimulation of mesothelial cells to secrete bFGF. We established mesothelial cell cultures from CHF patients with recurrent pleural effusions and incubated them with different concentrations of the talc. The cells produced significant levels of bFGF in a dose-dependent manner. These results support data of Antony et al. [20] who showed that talc can induce release of bFGF from mesothelial cells. Patients with higher pleural fluid bFGF levels after talc pleurodesis were more likely to develop successful pleurodesis. An increase in fibroblast proliferation was stimulated by incubation in pleural fluid from these patients, which was reduced by antibFGF antibodies. Antony et al. [20] demonstrated that talc pleurodesis is partly driven by mesothelial-derived bFGF. Furthermore, those authors demonstrated that patients with extensive pleural carcinomatosis and minimal intervening normal mesothelium had significantly lower quantities of bFGF in their pleural fluid compared to those with limited disease who subsequently developed successful pleural symphysis. These findings suggested that mesothelium free of tumor was necessary for successful pleurodesis [19]. A recent study also showed that an intact pleural mesothelium is critical in modulating the metastatic potential of cancer cells within the pleural space. Malignant cells secrete angiogenic factors that promote tumor growth, proliferation of endothelial cells and invasion of surrounding tissue by neovascularization. Talc-treated pleural mesothelium counteracts these effects by releasing endostatin, an anti-angiogenic factor which may be responsible for tumor containment within the pleural space and accounts for the improved clinical outcome of patients with malignant pleural effusions successfully pleurodesis with talc [24]. Lee et al. [25] demonstrated that talc causes apoptosis of lung cancer cells in a dose- and time-dependent manner. However, this process is selective and spares the normal mesothelium.

The mechanism that determines pleural symphysis involves the action of different growth factors, such as bFGF and TGF-*β* and, especially, vascular endothelial growth factor (VEGF). Ribeiro et al. [26] studied the acute effects of VEGF blockade on the expression of inflammatory cytokines and pleural fluid accumulation in rabbits that received intrapleural injections of either talc or silver nitrate. The animals pretreated with anti-VEGF antibody showed significant reductions in pleural fluid volumes after talc or silver nitrate injection. IL-8 levels, vascular permeability, and macroscopic pleural adhesion scores were also reduced in the groups that received bevacizumab. That study showed that bevacizumab interferes in the acute phase of pleural inflammation induced by silver nitrate or talc, reinforcing the role of VEGF as a key mediator in the production of pleural effusions.

Teixeira et al. [27] assessed the influence of the anti-VEGF antibody (bevacizumab) on pleurodesis induced by talc or silver in rabbits: antibody anti-VEGF interfered in the pleurodesis induced by both agents, and the anti-VEGF antibody inhibited adhesions between the pleural layers.

Collectively, our results provide experimental evidence that cytokine IL-8 levels were elevated during the early hours of pleural effusion after talc instillation and that this preceded the appearance of inflammatory cells in the pleural cavity. Profibrotic growth factor bFGF levels increased 24 hours after pleurodesis when fibroblasts started to proliferate in the pleura and produced collagen [19, 20], We also found that pleural mesothelial cells secrete both IL-8 and bFGF. Our present results closely agree with previous works of others that showed interactions among resident and inflammatory cells and cytokines and growth factors in the pathogenesis of tissue fibrosis during pleurodesis [18, 20, 22, 23]. These interactions lead to excessive matrix production with fibrosis and scar formation in the pleural cavity. Although fibroblasts have been implicated as the effector cells in pleural fibrosis, other resident cells, foremost among them, mesothelial cells, may directly or indirectly play important roles as well.

Pleural injury and fibrosis are characterized by excessive fibrin production. The pleural loculations evolve into fibrinous adhesions under the influence of profibrotic mediators, such as bFGF and TGF-*β*, leading to the deposition of collagen and the formation of adhesions between the visceral and parietal surfaces. Elucidation of the specific steps involved in inflammation and fibrin turnover in pleural injury during pleurodesis will lead to better understanding of this process and to the development of novel sclerosants with potential clinical applications.

At least two mechanistic pathways are likely to be involved in talc-induced pleural fibrosis: (i) the generation of cytokines and inflammation and (ii) the production of growth factors stimulating fibroblast proliferation and collagen synthesis. bFGF is considered to be the most potent profibrotic mediator, but the roles of other fibrogenic mediators need to be further studied as targets for pleurodesis. Although the mechanisms involved in pleural fibrosis are unclear, recent evidence suggests that they may be associated with the upregulation of genes for profibrotic mediators, such as bFGF and TGF-*β* [28]. However, further studies are required to establish a molecular mechanism in the development of pleural fibrosis.

In summary, talc pleurodesis is the consequence of sclerosant instillation and can manifest itself as diffuse pleural thickening and fibrosis. Although its pathogenesis is not completely known, it is likely that the complex interactions between resident and inflammatory cells and profibrotic mediators are integral to the development of pleural fibrosis. It is generally accepted that the primary target cells for pleural fibrosis are subpleural fibroblasts, however, our results as well as those of other studies suggest that mesothelial cells may also play a significant role in the pathogenesis of this condition, both by initiating inflammatory responses and producing profibrotic growth factors. A greater understanding of the biology of these growth factors may allow therapeutic manipulation of these cytokines to create pleurodesis and to stimulate pleural adhesion/fibrosis.
